# Glyoxylate, a New Marker Metabolite of Type 2 Diabetes

**DOI:** 10.1155/2014/685204

**Published:** 2014-11-27

**Authors:** Victoria J. Nikiforova, Pieter Giesbertz, Jan Wiemer, Bianca Bethan, Ralf Looser, Volker Liebenberg, Patricia Ruiz Noppinger, Hannelore Daniel, Dietrich Rein

**Affiliations:** ^1^Metanomics Health GmbH, 10589 Berlin, Germany; ^2^Timiryazev Institute of Plant Physiology, Russian Academy of Sciences, Moscow 127276, Russia; ^3^ZIEL Research Center for Nutrition and Food Sciences, Biochemistry Unit, Technische Universität München, 85354 Freising, Germany; ^4^metanomics GmbH, 10589 Berlin, Germany; ^5^Thermo Fisher Scientific, Clinical Diagnostics, BRAHMS GmbH, 16761 Hennigsdorf, Germany; ^6^Medizinische Fakultät Carl Gustav Carus, Technische Universität Dresden, 01307 Dresden, Germany

## Abstract

Type 2 diabetes (T2D) is characterized by a variety of metabolic impairments that are closely linked to nonenzymatic glycation reactions of proteins and peptides resulting in advanced glycation end-products (AGEs). Reactive aldehydes derived from sugars play an important role in the generation of AGEs. Using metabolite profiling to characterize human plasma from diabetic versus nondiabetic subjects we observed in a recent study that the reactive aldehyde glyoxylate was increased before high levels of plasma glucose, typical for a diabetic condition, could be measured. Following this observation, we explored the relevance of increased glyoxylate in diabetic subjects and in diabetic C57BLKS/J-Lepr^*db*/*db*^−/−^^ mice in the pathophysiology of diabetes. A retrospective study using samples of long-term blood donors revealed that glyoxylate levels unlike glucose levels became significantly elevated up to 3 years prior to diabetes diagnosis (difference to control *P* = 0.034). Elevated glyoxylate levels impact on newly identified mechanisms linking hyperglycemia and AGE production with diabetes-associated complications such as diabetic nephropathy. Glyoxylate in its metabolic network may serve as an early marker in diabetes diagnosis with predictive qualities for associated complications and as potential to guide the development of new antidiabetic therapies.

## 1. Introduction

Despite the long history of research on T2D, the knowledge about metabolic impairments and molecular mechanisms that participate in the development of diabetes is still limited. Central to diabetes pathology is a chronic hyperglycemia promoting the production of advanced glycation end-products (AGEs) that associate with inflammation and cause micro- and macrovascular damage [1] and with diabetic nephropathy, a severe and costly endpoint [2]. Preventing chronic hyperglycemia is the most critical goal of today's guidelines in preventing diabetes complications. Still, insulin resistance and insulin deficiency cause major impairments in the numerous metabolic pathways contributing to disease pathology.

Metabolite profiling approaches in plasma samples from subjects with impaired glucose tolerance and T2D revealed distinct changes in various metabolite classes [3–6]. Recently, Wang-Sattler et al. [7] described glycine and lysophosphatidylcholine levels as predictors for impaired glucose tolerance and T2D and those were associated with genetic variation in proteins of related pathways. Floegel et al. [8] identified increased serum hexose, phenylalanine, tyrosine, branched chain amino acids, and diacyl-phosphatidylcholine levels as closely associated with an increased risk of T2D. Wang et al. [9] described ketoacid derivatives of the branched chain amino acids as sensitive plasma indicators of insulin resistance and Sailer et al. [10] proposed citrulline to be part of obesity-dependent metabolic impairments. We focused on the identification of new marker metabolites of diabetes based on our metabolite profiling and discovery technology and found glyoxylate as a plasma marker that correlated with early diabetes in clinical studies [11]. Pathways that lead to glyoxylate production and utilization have been characterized by biochemists for more than a hundred years. Henry Drysdale Dakin (1880–1952), a pioneer in biochemistry, first investigated glyoxylic acid as an intermediate in mammalian metabolism [12]. Glyoxylic acid production was later demonstrated to derive from glycine [13] and from glycolate [14]. Its conversion into oxalate [15] and its retroconversion into glycolate [16] and glycine [17] were also described. Many years later the glyoxylate cycle, originally believed to be absent from mammalian metabolism, was hypothesized to play a role in human metabolic disease linking fatty acid overflow with glucose generation and insulin resistance [18].

The improved knowledge of these processes could help a better understanding of disease progression and strategies for interference. Animal models such as the C57BLKS/J-Lepr^*db*/*db*^−/−^^ mouse model, whose diabetic phenotype resembles diabetes mellitus in man [19], have been useful in exploring diabetes pathology. Here we further investigate glyoxylate in a mouse model and discuss the role of this aldehyde in the context of its metabolic network. Our results suggest that glyoxylate is a diabetes marker useful for further development towards improved diagnosis and novel treatment approaches.

## 2. Materials and Methods

### 2.1. Ethics Statement

Human experimental protocols of the studies herein described were approved by the responsible ethics commission and conducted according to the principles of the Declaration of Helsinki. All subjects participating in the sample collection study gave written informed consent. The address of the ethics commission was Ethik-Kommission der Bayerischen Landesärztekammer, Mühlbaurstrasse 16, 81677 München, Germany, approval from July 27th, 2008, number 08055. Animal care and use for this study was approved by the Veterinary Inspection Services, Section of Animal Protection, the Freising District Office, 85356 Freising, Germany (reference number: 32-568-2).

### 2.2. Prospective Clinical Study

Blood plasma samples were obtained from a cohort of the Bavarian Red Cross Blood Donation Service (Blutspendedienst des Bayerisches Rotes Kreuz, Munich, Germany) within a collaborative diabetes risk sample collection project cofunded by the “Bayerische Forschungsstiftung” (AZ 773-707). Overall 478 out of the 789 study participants were included in the metabolite profiling analysis of the prospective study part. Participants were categorized as newly identified diabetic subjects based on fasting plasma glucose (FPG) >7.0 mmol/L or by an oral glucose tolerance test (OGTT) with plasma glucose levels 2 hours after the standardized 75 g oral glucose challenge (2HPG) of ≥11.1 mmol/L [20]. Subjects at risk for diabetes were defined as those with impaired fasting glucose (IFG) levels of ≥5.6 to <7.0 mmol/L and those with impaired glucose tolerance (IGT) levels of ≥7.8 to <11.1 mmol/L. Nondiabetic control subjects had glucose levels below these threshold levels. Nondiabetic control subjects were matched for gender, age, and BMI to the diabetic subjects and subjects at risk for diabetes. Data sets were analyzed by computing ANOVA models corrected for confounders. All samples collected were processed according to metabolomics-specific standard operating procedures and stored at −80°C until analysis.

### 2.3. Retrospective Clinical Study

Plasma samples were obtained from a subset of 243 subjects of the prospective study described above for whom nonfasting plasma samples were available from previous routine blood donations at 0, 1.5, 3, and 6 years prior to study inclusion and diabetes diagnosis. Categorization of these subjects was done based upon FPG and OGTT data from the prospective study. Enrolled were *n* = 55 diabetic subjects according to FPG and/or OGTT criteria for diabetes [20]. The remaining subjects were categorized as with increased risk for diabetes (*n* = 92) and as nondiabetic subjects (*n* = 96) according to the same criteria. All samples collected retrospectively were processed according to standard operating procedures by the Bavarian Red Cross Blood Donor Service and stored at −42°C until transfer to Metanomics Health.

### 2.4. Type 2 Diabetes Mouse Model

Male mutant C57BLKS/J-Lepr^*db*/*db*^−/−^^ (*db*/*db* ^−/−^) mice and respective male C57BLKS/J wild-type controls (*n* = 6 for both groups) were purchased from Charles River Laboratories at an age of five weeks. All mice had ad libitum access to food and water throughout the whole feeding period, were conventionally housed with at least two animals per cage, and were analyzed at an age of 20 weeks in nonfasting state. For standardization, all mice were given the same chemically-defined control diet (ssniff EF R/M Kontrolle). Body weight was measured weekly and blood glucose was measured shortly before sacrifice. Animals were anesthetized with isoflurane, blood was collected into EDTA-coated tubes via retro-orbital puncture, and animals were sacrificed using cervical dislocation. All mice were sacrificed between 9:00 A.M. and 10:00 A.M. Tissues (liver and quadriceps muscle) were collected, snap-frozen in liquid nitrogen, and ground prior to extraction of metabolites. Metabolite profiling results of *db*/*db* ^−/−^ mice were analyzed against their black Kaliss (C57BLKS/J) wild-type control mice.

### 2.5. Metabolite Profiling

Two types of mass spectrometry analyses were applied to all samples: gas chromatography-mass spectrometry (GC/MS) and liquid chromatography-MS/MS for broad profiling as described previously [21]. Briefly, proteins were removed from plasma samples by precipitation prior to collection of polar and nonpolar fractions for both GC-MS and LC-MS/MS. For GC-MS analyses, the nonpolar fraction was treated with methanol under acidic conditions to yield the fatty acid methyl esters derived from both free fatty acids and hydrolyzed complex lipids. The polar and nonpolar fractions were further derivatized with O-methylhydroxylamine hydrochloride to convert oxo-groups to O-methyloximes and subsequently with a silylating agent prior to GC-MS analysis. For LC-MS/MS analyses, both fractions were reconstituted in appropriate solvent mixtures. High performance LC (HPLC) was performed by gradient elution on reversed phase separation columns. Mass spectrometric detection technology was applied as targeted and high sensitivity “multiple reaction monitoring” profiling in parallel to a full screen analysis. To account for inter- and intrainstrumental variation in GC-MS and LC-MS/MS profiling, data were normalized to the median of reference samples derived from a pool formed from aliquots of all samples. Pooled reference samples were run in parallel through the whole process. Key analytical parameters determined within method development and reviewed within the present study were taken into account to specify high quality metabolites, herein termed as semiquantitative metabolites.

Determination of the absolute concentration of glyoxylate in selected samples was performed by GC-SIM-MS in a single ion monitoring mode. The derivatized samples were separated by gas chromatography, and in the mass spectrometer three characteristic mass fragments were monitored. Quantification was performed with external calibration using stable isotopically labeled (^13^C_2_)-glyoxylate as internal standard. Accuracy was confirmed by spiking experiments with a concentration range of 0.55–80 *μ*mol/L spiked glyoxylate. Precision of glyoxylate quantification was 7%, limit of detection was 5.7 *μ*mol/L, and limit of quantification was 21.1 *μ*mol/L.

### 2.6. Statistical Analysis

Plasma samples from clinical studies were analyzed in a randomized sequence design with pooled samples (i.e., “pool”) generated from aliquots of each sample as a reference. Following comprehensive analytical validation steps, the raw peak data for each analyte was normalized to the median of the pool samples per analytical sequence to account for process variability (i.e., “pool-normalized ratios”). For identification of diabetes-associated differences between groups for each metabolite a univariate linear model was applied to the log-transformed pool-normalized ratios. The model was corrected for the fixed confounders age, gender, and BMI diabetes group. In the historic dataset, a mixed linear model including the owner ID as a random factor as well as the fixed factors time point of sample collection and the interaction between the time point of sample collection and diagnostic group in addition to the other confounders was calculated. Significances for differences in metabolite ratios between groups were assessed using *P* values of Students' *t*-statistics.

## 3. Results

### 3.1. Prospective Clinical Study

Fifty-eight participants were categorized as newly identified diabetic subjects based on FPG or 2HPG. The largest group consisted of subjects at risk for diabetes with IFG and/or IGT (*n* = 243), whereas *n* = 177 nondiabetic control subjects were included. Metabolomic marker discovery applying our metabolite profiling approach for diabetes screening revealed glyoxylate in addition to previously described metabolite markers as an early diagnostic metabolite. Curation of the profiling results confirmed glyoxylate elevated in diabetic and prediabetic subjects with a diabetic phenotype compared to controls (13%, *P* = 0.017) similar to the elevation of glucose (12%, *P* < 0.001) (Table 1).

### 3.2. Retrospective Clinical Study

In plasma samples collected up to 6 years prior to the diagnosis of diabetes glyoxylate increased earlier than glucose and thus may qualify as early diabetes marker (3 years prior, *P* = 0.034; 1.5 years prior, *P* = 0.081; at the last blood donation before study recruitment and diabetes classification, *P* = 0.036; Table 1). The rise in glyoxylate in subjects prior to diabetes diagnosis paralleled that of glucose but appeared earlier in the retrospective study and may thus be capable of predicting T2D earlier than plasma glucose. 

**Figure 1:**
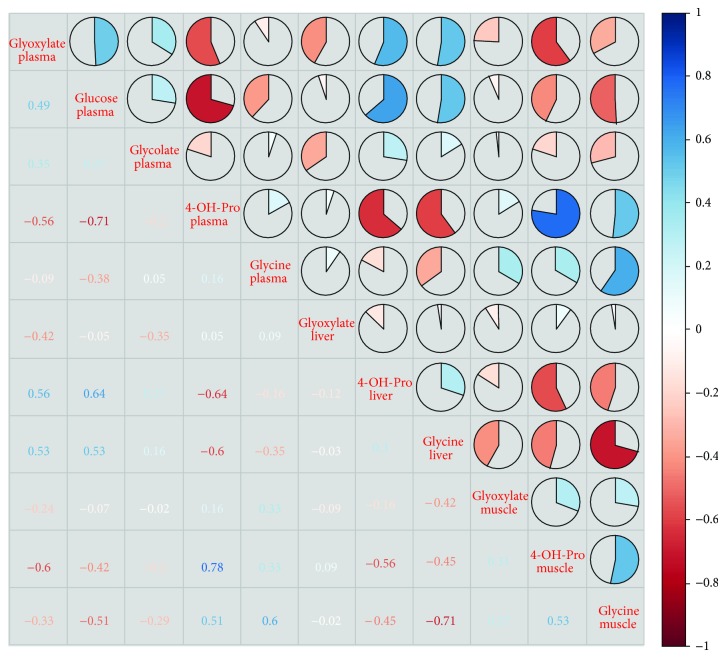
Correlation matrix with glyoxylate and precursors/products of glyoxylate metabolism. Kendall's tau correlations between metabolite levels of glyoxylate, glycine and 4-hydroxyproline in liver (L), muscle (M), and plasma (P) and of glycolate in plasma are visualized. Blue shades represent positive correlations and red shades represent negative correlations. The more the circle is filled, the higher the absolute correlation is.

*The C57BLKS/J-L* *ep* *r* ^*db*/*db*^−/−^^ *Diabetes Mouse Model.* C57BLKS/J-Lepr^*db*/*db*^−/−^^ (*db*/*db* ^−/−^) mice display a phenotype that resembles in many aspects that of humans with metabolic syndrome or diabetes characterized by hyperphagia, obesity, and hyperglycemia. Metabolite profiling revealed a 6-fold increase in plasma glyoxylate levels in *db*/*db* ^−/−^ mice as compared to wild-type animals (Table 2). Tissue metabolite profiling did not reveal a difference in liver glyoxylate levels but showed a small, yet significant, decrease in muscle glyoxylate levels (*P* = 0.025). Putative precursors or products of glyoxylate production and utilization with strong concentration changes were identified. Glycine was strongly decreased in liver and muscle tissue, but it was not significantly changed in plasma. 4-Hydroxyproline was strongly decreased in liver, muscle, and plasma. Correlation analysis, however, did not reveal robust associations between plasma or tissue levels of glyoxylate, glycine, or 4-hydroxyproline (see Figure 1). Also, no strong correlation was found between glyoxylate and glycolate in plasma. Significant correlations were only observed for 4-hydroxyproline between plasma and tissues.

**Figure 2:**
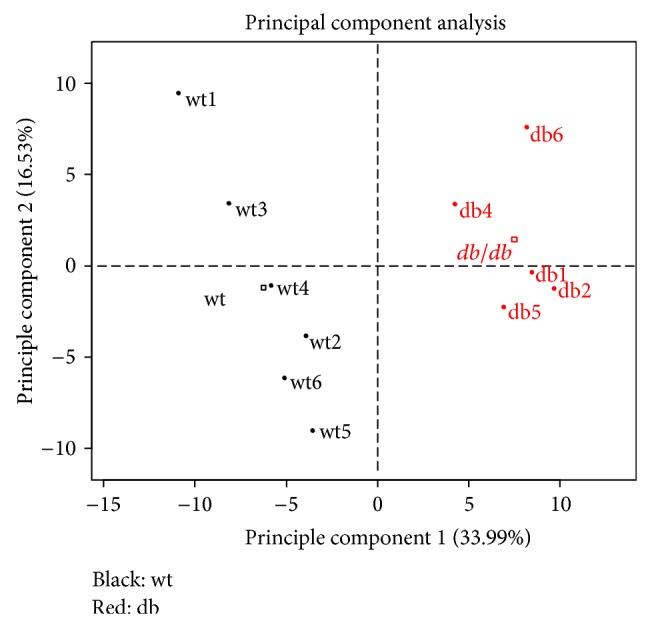
Principal component analysis separating individual mice. Principal components are calculated based on plasma metabolite levels (wt: wild-type mice, db: *db*/*db* ^−/−^ mice).

**Figure 3:**
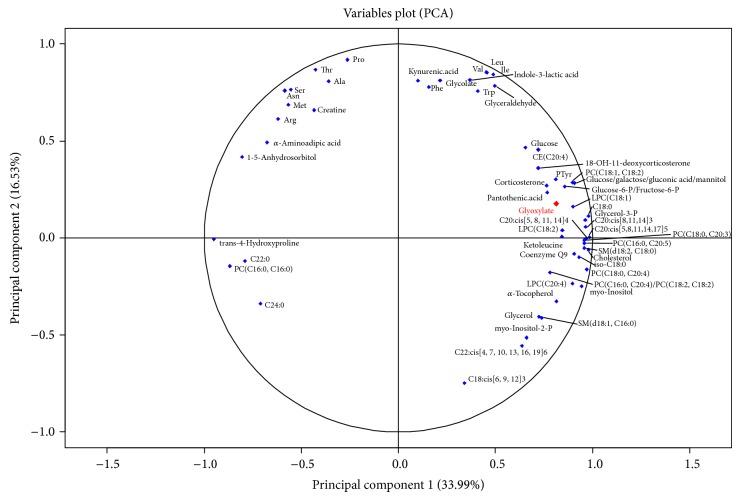
Variables plot showing plasma metabolites responsible for separation of individual mice. To visualize the strongest separators only, square correlations from both dimensions are summed for all metabolites and metabolites with a minimal sum of 0.6. The more distant the metabolites are from the circle center, the higher the correlation with the dimension (PC: phosphatidylcholine, LPC: lysophosphatidylcholine) is.

Principal component analysis of plasma metabolites however shows clear separation of wild-type and *db*/*db* ^−/−^ mice in the first dimension (Figure 2). Metabolites which are strong separators for both mouse groups are visualized in the variables plot shown in Figure 3. Glyoxylate is a strong separator of wild-type and *db*/*db* ^−/−^ mouse groups showing a high positive correlation with the first dimension. A large number of other metabolites previously described as associated with diabetes are also found here as strong separators of wild-type and *db*/*db* ^−/−^ mice including for example glucose, 1,5-anhydrosorbitol [22], and 2-amino adipic acid [9], as well as the leucine-derivative alpha-ketoisocaproic acid (ketoleucine) [9].

## 4. Discussion

Metabolite profiling approaches have revealed a set of plasma and urinary metabolites that change when healthy people move into insulin resistance or diabetes. With the findings of the present study we add glyoxylate—a two-carbon reactive aldehyde—to the growing list of marker metabolites of diabetes. In patients with newly diagnosed diabetes, glyoxylate was found to associate with diabetes development and changes in glyoxylate plasma levels occur already three years prior to the diagnosis of diabetes or prediabetes. Increased plasma glyoxylate levels were also observed in the *db*/*db* ^−/−^ mouse model supporting the results that glyoxylate is associated with the diabetic phenotype. Glyoxylate metabolism has been studied in cell cultures, animals, and humans [23, 24]. To further investigate the source of high glyoxylate plasma concentrations in the clinical diabetic and prediabetic stages, we confirmed strongly elevated plasma levels in *db*/*db* ^−/−^ mice. Analysis of liver and muscle tissue could not identify the origins for the change in glyoxylate level observed in plasma. Moreover, a clear contribution of glyoxylate precursors glycolate, glycine, and 4-hydroxyproline to the increase in plasma glyoxylate levels could not be determined.

**Figure 4:**
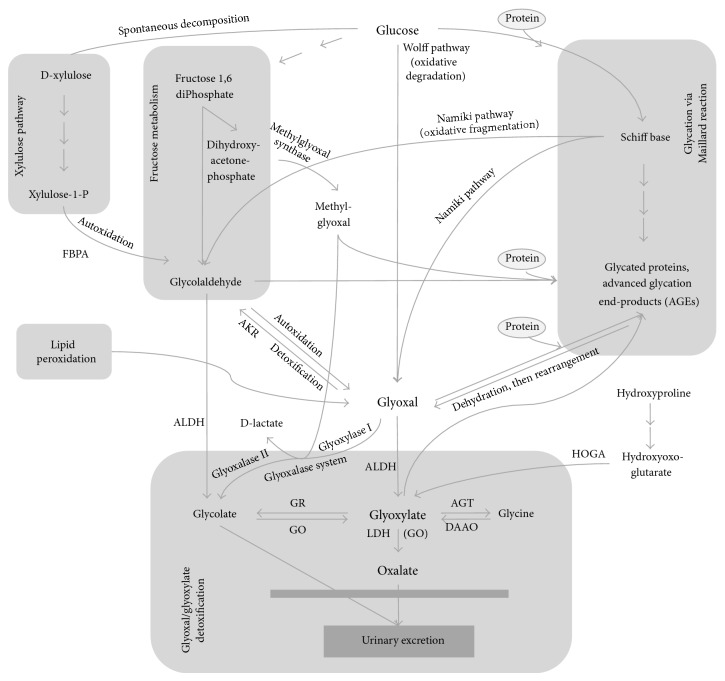
Glyoxylate biochemical pathways for mammals. AGT: alanine-glyoxylate aminotransferase; AKR: aldo-keto reductase; ALDH: aldehyde dehydrogenase; DAAO: D-amino acid oxidase; FBPA: fructose-bisphosphate aldolase; GO: glycolate oxidase; GR: glyoxylate reductase; HOGA: 4-hydroxy-2-oxoglutarate aldolase; LDH: lactate dehydrogenase; PFK: phosphofructokinase.

Pathways that lead to the production of glyoxylate are similar in microorganisms, fungi, plants, and some invertebrates where the glyoxylate cycle is the prime source for the aldehyde during gluconeogenesis. In humans and higher organisms, glyoxylate is mainly a product of enzymatic glycolate oxidation in peroxisomes (Figure 4). This pathway is well-characterized as it is impaired in primary hyperoxaluria with abnormally increased production of oxalate for which glyoxylate is the major and direct precursor [25].

Glyoxylate is also a mitochondrial degradation product of hydroxyproline, which is derived from the dietary meat or from endogenous collagen breakdown [26]. Diabetic *db*/*db* ^−/−^ mice in our study showed very high glyoxylate levels in plasma but depressed levels in muscle tissue indicating that muscle turnover or intramuscular glucose were not significant sources of glyoxylate. Another endogenous source of glyoxylate is glyoxal, which can be converted to oxalate [27], and glyoxylate has also been shown to be a product of glycine deamination catalyzed by kidney D-amino acid oxidase [13, 28]. In the *db*/*db* ^−/−^ mice, we found strong decreases compared to the wild-type mice for hydroxyproline in plasma, liver, and muscle and for glycine in liver and muscle. However, the lack of strong correlations to plasma and tissue glyoxylate levels suggests that these metabolites do not directly contribute to changes in plasma glyoxylate levels.

Interestingly, some precursors of glyoxylate are closely related to glucose metabolism products. Evidence that glucose can be converted into glyoxylate was provided by experiments in HepG2 cells [27] which produce ^13^C_2_-labeled glycolate and ^13^C_2_-labeled oxalate from ^13^C_6_-labeled glucose. HepG2 cells can also convert glyoxylate into oxalate [29]. This feature of liver cells to derive glyoxylate from glucose may explain the correlation between plasma glucose and glyoxylate levels.

Glyoxylate is involved in major metabolic pathways; still, it has only been detected as a low abundant metabolite. We measured glyoxylate concentrations in human plasma of around 25 *μ*mol/L in healthy subjects and >30 *μ*mol/L in T2D subjects. Glyoxylate measured in human liver samples was as low as 128 pmol/mg protein and in HepG2 cells around 38 pmol/mg protein corresponding to 6 *μ*mol/L based on cell volume [29] suggesting that there is a regulation of cellular glyoxylate concentrations [30] allowing glyoxylate efflux into blood despite higher extracellular concentrations.

Amongst the pathways in which glyoxylate is metabolized, the conversion to glycine catalyzed by alanine-glyoxylate aminotransferase (AGT) in liver peroxisomes dominates. A second pathway is the oxidation of glyoxylate to oxalate by lactate dehydrogenase (LDH) with oxalate as a final metabolite in mammals. The third pathway is the enzymatic reduction of glyoxylate to glycolate by cytosolic glyoxylate reductase (GR), which occurs predominantly in the liver. Finally, the products oxalate and glycolate are then excreted with the urine [31].

It is well established that elevated plasma glucose levels cause damage of proteins by glycation and formation of AGEs. Most AGEs arise from reaction of the carbonyl group of reducing sugars such as glucose or from short-chain aldehydes with amino groups of proteins, lipids, and nucleic acids. Protein glycation reactions cause dysfunctions associated with various diseases in particular cardiovascular disease [32, 33] and alpha-oxoaldehydes, such as glyoxal, methylglyoxal, and 3-deoxyglucosone which are important precursors of advanced glycation adducts [34].

Glyoxal can be derived from oxidative fragmentation of a protein-sugar Schiff base, also known as the Namiki pathway [34], by oxidative degradation (autoxidation) of glucose via the Wolff pathway [35, 36] or by degradation of glycated proteins and AGEs [37] (Figure 4). In addition, it can derive from lipid peroxidation reactions [38–40], the autoxidation of glycolaldehyde [41], and other reactive intermediates within the Maillard reaction [42]. Glyoxal is also a by-product of fructose and xylulose metabolism [27, 43].

*α*-Oxoaldehydes are potent glycating agents with reactivity several thousand-fold higher than glucose in causing AGEs formation [44]. The reactivity of glyoxylate towards the production of AGEs has been shown with lysine, arginine, and glucosamine [45]. Compared to reducing sugars, glyoxylate was 60% more reactive than glucose or fructose and 20% more reactive than glyceraldehydes [45]. Glyoxylate also reacted when incubated with human serum albumin (HSA) producing approximately 10% of lysine and 20% of arginine side chain modifications. The fibrillar state as a measure of aggregation of a glyoxylic acid-modified HSA was shown to be enhanced, suggesting conformational changes of the protein to a degree of reduced biological functionality [46]. Thus, in addition to the well-known reactivity of glyoxal and methylglyoxal [47], glyoxylate appears to be involved in high glucose-induced protein damage by advanced glycation.

This calls for proper detoxification mechanisms in control of *α*-oxoaldehydes. Detoxification of glyoxal is primarily achieved by the glyoxalase system which consists of glyoxalase I, glyoxalase II, and glutathione [48]. It detoxifies *α*-oxoaldehydes to the corresponding aldonic acid. The product of glyoxal detoxification by the glyoxalase pathway is glycolate [49, 50], a substrate for glycolate oxidase (GO) to produce glyoxylate [51] (Figure 4). The glyoxalase system also detoxifies methylglyoxal to lactate.

An important second pathway is the detoxification of the AGE precursor glyoxal by aldo-keto reductases [51, 52] leading to glycolaldehyde, which can be converted to glyoxylate via glycolate by aldehyde dehydrogenase (ALDH) and glycolate oxidase (Figure 4). Aldehyde dehydrogenase is also involved in the third detoxification pathway that directly metabolizes the carbonyl substrate glyoxal in an irreversible enzymatic oxidation reaction to glyoxylate. The appropriate enzyme was isolated from human liver and demonstrated to be involved in the removal of aldehydes [53]. All these pathways of glyoxal detoxification produce glyoxylate, either directly or indirectly, and thus link the changes in observed glyoxylate levels to pathways and mechanisms central to the pathophysiology of diabetes.

An important pathophysiological concept in the context of reactive aldehyde changes observed in this metabolite profiling study is the AGEs induced diabetic nephropathy [54, 55]. Chronic hyperglycemia is associated with diabetic nephropathy, and AGEs have been shown to be involved in its development and progression [56]. Glyoxylate may be directly and/or indirectly implicated in AGE mediated renal metabolic disorders. Direct implication would be by reacting with its *α*-oxoaldehyde group, indirectly by contributing a sensitive indicator of a glycating burden that accumulates in blood and causes damage to renal vessels.

Another aspect of diabetes-associated renal metabolic disorders to be reconsidered in light of glyoxylate is the implication of oxalate nephropathy. The findings that the direct oxalate precursor glyoxylate is at the same time a product of glyoxal detoxification provide a causal link between accumulation of AGEs and oxalate induced nephropathy leading to chronic renal failure, connecting AGEs mechanistically to renal pathophysiology. Indeed, association between diabetes and increased excretion of urinary oxalate was shown [57], and the finding that such increase is independent of the dietary oxalate intake gave rise to the hypothesis that it is due to diabetes pathophysiology [58]. However, recent clinical studies on the chemical composition of urinary stones did not reveal direct associations between oxalate stones and metabolic syndrome [59, 60].

Based on our metabolite profiling approaches in a mouse model of diabetes and in human samples and in view of the scientific literature investigating the production, utilization, and role of glyoxylate, we have placed the new metabolic marker glyoxylate in context of diabetes pathology. The strength of this new marker is its link to protein-modifications causing long-term diabetes compilations. Glyoxylate can thus be considered as “diabetes-metabolite” with predictive biomarker-quality.

## Figures and Tables

**Table 1:** Increased plasma glyoxylate levels allow for diabetes diagnosis earlier than plasma glucose. Glyoxylate is significantly increased in blood plasma in type 2 diabetes and starts to rise in nonfasted subjects later diagnosed as prediabetic or diabetic up to 3 years prior to T2D diagnosis. For comparison, corresponding data on glucose changes from the same samples are provided. Significant differences in glyoxylate levels were detectable in fasting as well as in nonfasting plasma samples. Fasting plasma was obtained from diabetes and diabetes risk subjects (*n* = 301) and from age, gender, and BMI matched nondiabetic subjects (*n* = 177) participating in the prospective study; retrospective analyses of samples available (or stored) at the biobank of the Blood Donation Service were from diabetes and diabetes risk subjects (*n* = 147) and from nondiabetic subjects (*n* = 96) of the above described study group.

Study condition		Fasting plasma	Nonfasting plasma			
Years prior to diagnosis of diabetes and diabetes risk		0	0	1.5	3	6
Glucose	Ratio diabetes and diabetes risk versus nondiabetic subjects	**1.12** ^*^	1.08^***^	1.08^***^	1.07	1.01
	(*P* value)	<0.001	0.059	0.060	0.106	0.810

Glyoxylate	Ratio diabetes and diabetes risk versus nondiabetic subjects	1.13^**^	1.21^**^	1.17^***^	1.21^**^	1.03
	(*P* value)	0.017	0.036	0.081	0.034	0.719

^*^ *P* < 0.01, ^**^ *P* < 0.05, ^***^ *P* < 0.10.

**Table 2:** Comparison of *db/db* ^−/−^ and wild-type mice for plasma and tissue levels of glyoxylate and metabolites involved in glyoxylate synthesis or degradation (n.s.: not significant and FC: fold-change *db/db* ^−/−^ relative to wild-type).

Metabolite	Plasma		Liver		Quadriceps muscle	
	FC	*P* value	FC	*P* value	FC	*P* value
Glucose	2.15	*P* < 0.01	1.39	*P* < 0.01	2.23	*P* < 0.01
Glyoxylate	6.06	*P* < 0.001	1.05	n.s.	0.67	*P* < 0.05
Glycolate	1.13	n.s.	Not detected		Not detected	
Glycine	0.81	n.s.	0.62	*P* < 0.001	0.49	*P* < 0.001
4-Hydroxyproline	0.26	*P* < 0.001	0.26	*P* < 0.001	0.14	*P* < 0.001
